# Algorithm for the resuscitation of traumatic cardiac arrest patients in a physician-staffed helicopter emergency medical service

**DOI:** 10.1186/cc12219

**Published:** 2013-03-19

**Authors:** PB Sherren, C Reid, K Habig, B Burns

**Affiliations:** 1 Greater Sydney Area HEMS, Sydney, Australia

## Introduction

Survival rates following traumatic cardiac arrest (TCA) are known to be poor but resuscitation is not universally futile [1]. There are distinct differences in the pathophysiology between medical cardiac arrests and TCA. Traumatic pathologies associated with an improved chance of successful resuscitation include hypoxia, tension pneumothorax and cardiac tamponade [1]. The authors believe a separate algorithm is required for the management of out-of-hospital TCA attended to by a highly trained physician and paramedic team.

## Methods

A suggested algorithm for TCA was developed based on the Greater Sydney Area Helicopter Emergency Medical Service's standard operating procedures and current available evidence.

## Results

**Figure 1:**
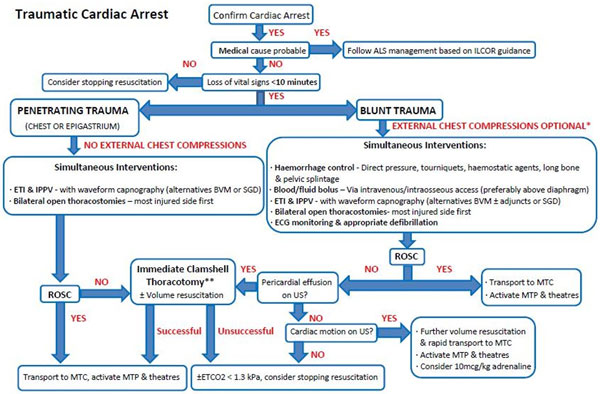
**Traumatic cardiac arrest algorithm**.

**Figure 2:**
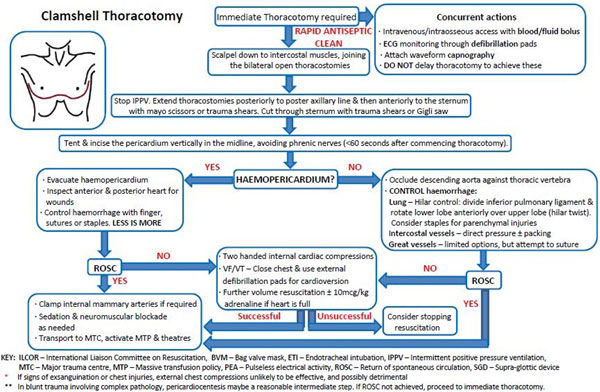
**Thoracotomy algorithm**.

An algorithm for the general management of TCA can be seen in Figure 1. In TCA, priority should be given to catastrophic haemorrhage control (tourniquets, direct pressure, haemostatic agents, pelvic and long bone splintage) and volume resuscitation. Simultaneous oxygenation optimisation should occur with proactive exclusion of tension pneumothoraces with bilateral open thoracostomies. Cardiac ultrasound (US) should be used to help exclude cardiac tamponade and assist in prognostication. The US presence of true cardiac standstill versus low pressure state/pseudo-PEA, and an ETCO_2 _<1.3 kPa carries a grave prognosis in TCA. Given the high incidence of hypovolaemia, hypoxia and obstructive shock prior to TCA, the role of adrenaline and chest compressions are limited. Figure 2 shows a suggested algorithm for the management of penetrating TCA requiring prehospital thoracotomy.

## Conclusion

The suggested algorithm is designed for a highly trained physician-led prehospital team and aims to maximise the number of neurologically intact survivors in out-of-hospital TCA.
